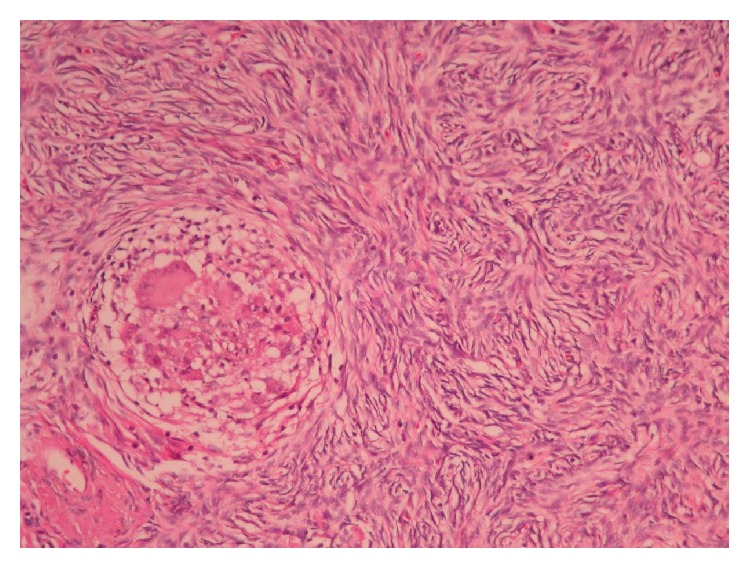# Abdominal Sarcoidosis May Mimic Peritoneal Carcinomatosis

**DOI:** 10.1155/2015/263945

**Published:** 2015-10-08

**Authors:** Umit Gorkem, Tayfun Gungor, Yılmaz Bas, Cihan Togrul

**Affiliations:** ^1^Department of Obstetrics and Gynecology, Hitit University Faculty of Medicine, 19040 Çorum, Turkey; ^2^Department of Pathology, Hitit University Hospital, 19040 Çorum, Turkey

## Abstract

Sarcoidosis is a multisystem inflammatory disorder of unknown etiology. It shows a great variety of clinical presentation, organ involvement, and disease progression. Lungs and lymphoid system are the most common sites involved with a frequency of 90% and 30%, respectively. Extrapulmonary involvement of sarcoidosis is reported in 30% of patients and abdomen is the most frequent site. Furthermore, peritoneal involvement is extremely rare in sarcoidosis. The case presented here described peritoneal manifestations of sarcoidosis without involvement of lungs. A 78-year-old woman possessing signs of malignancy on blood test and abdominal magnetic resonance imaging underwent laparatomy with a suspicion of ovarian malignancy. The macroscopic interpretation during surgery was peritoneal carcinomatosis. Total abdominal hysterectomy, bilateral salpingo-oophorectomy, peritoneal biopsies, total omentectomy, and appendectomy were performed. Final histopathological result revealed the diagnosis of sarcoidosis. Clinicians must keep in mind that peritoneal sarcoidosis can mimic intra-abdominal malignancies.

## 1. Introduction

Sarcoidosis is a multisystem inflammatory disorder of unknown etiology. The cause may be related to a bacterial or fungal infection or an immune response from an unidentified antigen [1]. The genetic factors may be important for the risk of disease as well as its pattern, severity, and prognosis [2]. It shows a great variety of clinical presentation, organ involvement, and disease progression. Noncaseating granulomas in the absence of other granulomatous diseases is the characteristic for sarcoidosis [3]. Sarcoidosis is usually diagnosed between 20 and 40 years of age. Women are more frequently affected than men. The etiology of the disease is still not exactly clarified. Lungs and lymphoid system are the most common sites involved with a frequency of 90% and 30%, respectively [4, 5]. Extrapulmonary involvement of sarcoidosis is reported in 30% of patients and abdomen is the most frequent site. Furthermore, peritoneal involvement is extremely rare in sarcoidosis [6]. Abdominal sarcoidosis can occur in the absence of lymphatic or pulmonary disease [7]. Although usually asymptomatic, the presence of symptomatic abdominal involvement may affect the prognosis and treatment options. Symptomatic abdominal sarcoidosis requires treatment with immunosuppressant agents. Surgical interventions may be required in the presence of gastrointestinal complications such as massive hemorrhage, strictures, obstruction, or perforation. Splenectomy can be performed for symptomatic relief in splenic involvement or as prophylaxis for splenic rupture. Cardiopulmonary involvement is the main cause of death. Here we present a rare case of peritoneal sarcoidosis mimicking peritoneal carcinomatosis.

## 2. Case Report

**Figure 1:**
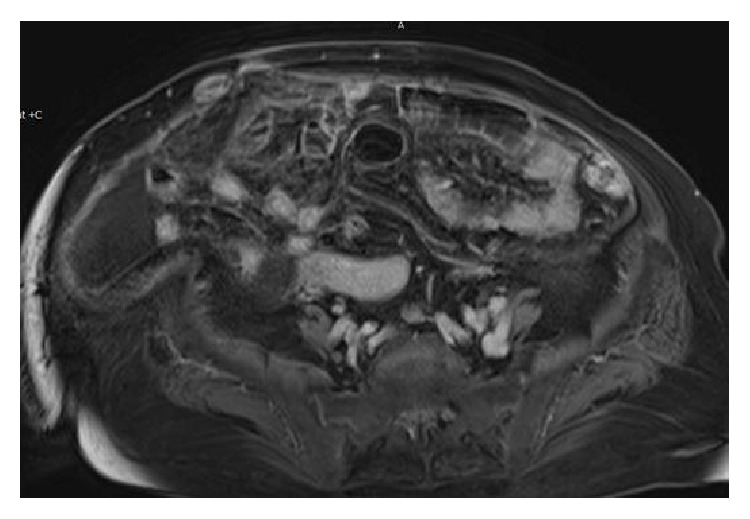


**Figure 2:**
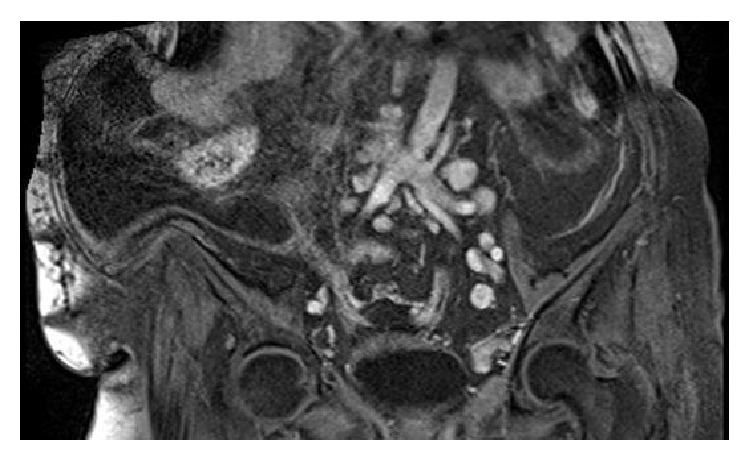


**Figure 3:**
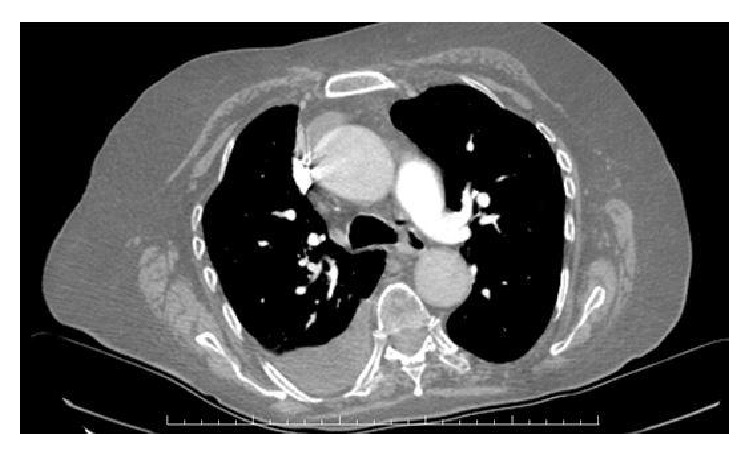


A 78-year-old woman, gravida 4, para 3, presented with complaints of dyspnea, abdominal discomfort, and distension that persisted for one month on December 2014. Her medical history revealed no history of tuberculosis and of asbestos exposure. On abdominal examination, she had abdominal tenderness without rebound or defense signs. Hepatosplenomegaly and lymphadenopathy were not noted on physical examination. Chest examination was dull to percussion over both hemithorax and respiratory sounds diminished at both basal regions. Although blood tests were at normal range, serum CA 125 and CA 15.3 levels were 116 u/mL and 49,1 u/mL, respectively. Ultrasonography of the abdomen confirmed the presence of diffuse ascites. Chest radiography revealed bilateral pleural effusion. Thickening of parietal peritoneum and nodular implantation at right side of lower abdomen and sign of omental cake were detected on T1-weighted fat-saturated, contrast-enhanced transverse images of abdominal magnetic resonance (MR) (Figure 1). T1-weighted fat-saturated, contrast-enhanced coronal images of MR also showed several lymphadenopathies (LAPs) that were measuring maximally 52 × 28 mm in diameter accompanied by main abdominopelvic vessels (Figure 2). Contrast-enhanced computerized tomography (CT) of the chest also demonstrated no pathological finding except bilateral pleural effusion (Figure 3).

An explorative laparotomy procedure was planned with the preliminary diagnosis of intraabdominal malignancy. At laparotomy under spinal anesthesia, miliary white nodules measured 2–5 mm in diameter studded the diaphragm, liver, omentum, gastric serosa, small and large intestines, whole surface of parietal peritoneum, and both ovaries. Furthermore, approximately 1,5 liters of nonbloody ascites was aspirated from intraabdominal cavity. The sample of ascites was sent for cytological examination. Omental cake appearance was prominent. Interpretation of case was manifestation of peritoneal carcinomatosis. Cytological examination and frozen section pathology resulted in the fact that malignancy could not be excluded. Then we performed total abdominal hysterectomy, bilateral salpingo-oophorectomy, peritoneal biopsies, total omentectomy, and appendectomy. Numerous lymphadenopathies of bilateral iliac, obturator, and paraaortic sites were noted. The size of lymph nodes ranged from 2,0 cm to 5,5 cm in diameter. Enlarged lymph nodes were sampled during the operation. She was discharged from the hospital after uneventful postoperative course at 10th day.

Final histopathology demonstrated epithelioid noncaseating granulomas containing multinucleated giant cells in the serosal surfaces of uterus, ovaries, fallopian tubes, peritoneum, and omentum as well as iliac, obturator, and paraovarian lymph nodes (Figure 4). The patient was referred to the department of pulmonary medicine. She had a negative tuberculin skin test. The diagnosis was consistent with extrapulmonary sarcoidosis without pulmonary involvement. A course of steroid therapy was started. Currently, the patient is doing well and is followed up by the departments of gynecologic oncology and pulmonary medicine.

## 3. Discussion

The manifestations of abdominal sarcoidosis are less characteristic. It may mimic neoplastic or infectious diseases such as lymphoma, diffuse metastasis, granulomatous, and mycobacterial infection [8]. The other nonneoplastic conditions include systemic diseases such as eosinophilic gastroenteritis and amyloidosis, tumour-like conditions such as aggressive fibromatosis or inflammatory pseudotumors, Whipple disease, endometriosis, and actinomycosis [9]. Peritoneal sarcoidosis should be considered in the differential diagnosis with tuberculosis even in the context of a negative PPD, fungal infections, and carcinomatosis [10]. Laparotomy or laparoscopy is almost always essential to demonstrate the involvement of the disease. Peritoneal biopsy is also needed for the differential diagnosis of sarcoidosis.

Exudative ascites (bloody and nonbloody) and abdominal pain are the mostly seen clinical presentations. Multiple granulomatous nodules involving peritoneum or a single peritoneal lesion may be seen. Enlarged lymph nodes are detected in nearly 30% of patients, especially in the porta hepatis, para-aortic region, and the celiac axis [11]. Interestingly, LAPs in our case were prominent in para-aortic, iliac, and obturator regions. Unlike lymphoma, enlarged lymph nodes are smaller than 2 cm in size and more scattered. Involvement of retrocrural area is less common in the cases of lymphoma [12]. The LAPs in the presented case ranged from 2,0 cm to 5,5 cm in diameter.

Omental cake is an unfavorable sign and mostly caused malignancy. It is associated with infiltration of the omental fat. The other causes of omental cake are inflammatory conditions such as tuberculosis, Crohn's disease, phlegmonous pancreatitis, and granulomatous enterocolitis. The differential diagnosis should also be focused on benign tumors derived from lymphatic, vascular, neuromuscular, or fatty tissues [13]. To our surprise, the source of omental cake in our case was sarcoidosis.

Homogenous hepatosplenomegaly (HSM) is typically related to extrapulmonary sarcoidosis. In 5 to 15% of cases, multiple, hypoechoic, hypodense, and hypointense nodules are scattered in the liver and spleen. HSM in sarcoidosis is easily misdiagnosed as lymphoma, infection, or metastatic disease [14].

Sclerosing peritonitis is a syndrome characterized by abdominal pain, intestinal obstruction, and thickening of peritoneum with massive adhesions. This condition is easily curable by steroid therapy. The other causes of sclerosing peritonitis are bacterial infections (mycobacteria), foreign bodies, peritoneal dialysis, asbestos exposure, carcinoid syndrome, or being idiopathic [15]. Almost all manifestations except intestinal obstruction exist in the presented case.

Carbohydrate antigen or cancer antigen 125 (CA 125) is a tumor marker secreted by the mesothelial cells lining the peritoneal cavity. It has also been identified on tissues of nonmesothelial origin such as tracheobronchial epithelium, epithelium of pancreas, colon, gallbladder, stomach, kidney and breast, amniotic tissue, placenta, and cervical mucous membrane. Therefore, inflammation of these regions can yield elevated serum levels of CA 125. Elevated CA 125 levels have also been reported in endometriosis, pelvic inflammatory disease, during menstruation and pregnancy, after laparotomy, and in numerous conditions [16]. Interestingly, the molecule is not evident on the surface of normal ovarian cells but evident on malignant nonmucinous ovarian cells [16]. Besides, CA 125 at low concentration is determined in healthy females and males. Klug et al. have suggested that elevated CA 125 levels may be derived from the “presence of serum factors other than the defined antigenic determinants” [17]. Peritoneal involvement of sarcoidosis may be reflected by elevation of serum CA 125 level. It is not well known whether CA 125 would be a reliable marker for sarcoidosis activity.

Although isolated occurrence of sarcoidosis in the genital system is rare, uterine and ovarian involvement are most common sites relatively. Like uterine sarcoidosis, most of the ovarian ones are reported in the reproductive age period. A few cases of ovarian sarcoidosis in postmenopausal women were reported as in our case presentation [18]. Symptoms are usually nonspecific and presented with clinical features concerning ovarian tumors. Finding of sarcoid-like lesions may mask an coexisting malignancy [19]. The involvement of the fallopian tubes mostly occurs in conjunction with sarcoidosis of the other parts of female genital system.

In conclusion, sarcoidosis should be kept in mind for differential diagnosis of intraabdominal malignancies that can affect female genital organs and peritoneum. Therefore, optimum management of such patients should be delivered and inessential invasive procedures can be avoided.

## Figures and Tables

**Figure 4:**